# Sex and recombination in aflatoxigenic Aspergilli: global implications

**DOI:** 10.3389/fmicb.2014.00032

**Published:** 2014-02-05

**Authors:** Geromy G. Moore

**Affiliations:** Southern Regional Research Center, Agricultural Research Service, United States Department of AgricultureNew Orleans, LA, USA

**Keywords:** *Aspergillus*, aflatoxin, sexuality, recombination, climate change, biocontrol

## Abstract

For most of the half century that aflatoxigenic species have been intensively studied, these molds were known only to reproduce asexually, with parasexuality found only in the laboratory between certain mutant strains. Therefore, the fairly recent discovery of their sexual (teleomorphic) states creates a new wrinkle in our understanding of the field behavior of these agriculturally significant fungi. Sex within populations of these fungi, and attendant genetic recombination, eventually may create difficulties for their control; and subsequently for the protection of important human and animal food supplies. Moreover, if fungal sex is a form of response to ecological and environmental stressors, then perhaps human influence and climate change could accelerate this phenomenon. This article will explore scientific research into sexuality and recombination in aflatoxigenic *Aspergillus* species; the potential impacts these phenomena could have on a popular method of pre-harvest prevention of aflatoxin contamination (i.e., use of non-aflatoxigenic *A. flavus* for biocontrol); and the outlook for maintaining control of aflatoxin contamination in an era of changing global climate.

## INTRODUCTION

Within the expansive community of agriculturally significant fungi are species that pose health risks to animals and humans through the production of mycotoxins; consequently, these mycotoxigenic fungi are important to understand and to control (Bennett and Klich, 2003). Aflatoxins are the most serious agricultural mycotoxins. They are mostly produced by a group of fungal species within the genus *Aspergillus*’ section *Flavi *(Bennett, 2010), although a few other species outside of this section produce aflatoxins, and carry homologs of the genes for aflatoxin (AF) synthesis (Bradshaw, 2006; Cary et al., 2009; Bradshaw et al., 2013). *Aspergillus flavus* and *A. parasiticus *are the most prominent aflatoxigenic (AF+) species with agricultural significance, contaminating cereal and oilseed crops as well as tree nuts; *A. nomius* has also been reported in agricultural fields (Feibelman et al., 1998; Ehrlich et al., 2007). The global economic losses due to contamination by these fungi are in the billions of dollars (Wu et al., 2008). AF contamination causes negative impacts on health and life across the globe, especially in many developing nations where inhabitants lack the education regarding the risks of consuming aflatoxin-contaminated foods, lack understanding of the importance for proper food storage, or perhaps would rather risk eating contaminated crops than go hungry (Williams et al., 2004; Shephard, 2008). The burden of illness and death associated with aflatoxin consumption is a constant reminder that efficient AF control measures are in need of globalization.

Researchers are working to develop a food supply that is free of AF contamination through implementation of pre-harvest and/or post-harvest strategies (Hell et al., 2008; Abbas et al., 2011). One of the most appealing control measures involves the use of naturally occurring, non-aflatoxigenic (AF-) *A. flavus* as pre-harvest biological control instead of chemical fungicides. This method involves the field dispersal of a high volume of inocula composed of a highly competitive AF- strain. The presence of the AF- strains interferes with the proliferation of indigenous AF+ fungi (Abbas et al., 2011). Studies on the genetic background of these AF- strains show that they result from either random mutations in, or absence/loss of, genes necessary for AF synthesis (Ehrlich and Cotty, 2004; Chang et al., 2005; Moore et al., 2009). Since their introduction a decade ago, two commercially available biopesticides have been in active use throughout the United States known as AF36 and Afla-Guard^®^. Currently, other strains are being studied and developed as potential biocontrol agents, not only in the U.S. but also in other parts of the world (Pitt and Hocking, 2006; Abbas et al., 2011; Probst et al., 2011). This method of control is gaining favor due to its proven success at reducing AF contamination in the field, but our confidence in using AF- *A. flavus* strains as biopesticides is based largely on the logic that they are predominantly asexual and genetically stable (Ehrlich and Cotty, 2004).

## RECOMBINATION AND SEXUALITY IN SECTION *FLAVI*

Ken Papa explored parasexual recombination in *A. flavus* and *A. parasiticus* 40 years ago, but his findings were limited to laboratory experiments between mutant strains (Bennett, 1985). David Geiser was one of the first to report evidence for genetic recombination in *A. flavus,* due to a cryptic sexual state, and hence the potential risk for using *A. flavus* strains as biocontrol (Geiser et al., 1998). His findings inspired further studies by Ignazio Carbone’s research group to uncover supportive evidence of recombination within the AF gene clusters of *A. parasiticus* and *A. flavus* populations sampled from within the same peanut field in the U.S. (Carbone et al., 2007; Moore et al., 2009). Recombination breakpoints within the AF gene clusters of *A. parasiticus* and *A. flavus* were observed, even though the *A. parasiticus* population exhibited less historical recombination compared to the *A. flavus* population in the same field (Moore et al., 2009). Moore et al. performed a global scale study of recombination for *A. flavus* and *A. parasiticus* field populations, representing five separate continents, and observed evidence of recombination within each *A. flavus* population examined and for two of the *A. parasiticus* populations (Moore et al., 2013a). Similar patterns of linkage disequilibrium (LD) could be observed for global *A. flavus* L-strain populations, although the same could not be observed for all of the sampled *A. parasiticus* or *A. flavus* S-strain populations. Ramirez-Prado et al. (2008) characterized mating alleles in *A. flavus* and *A. parasiticus*, and determined that since only one mating-type idiomorph (*Mat1-1* or *Mat1-2*) could be amplified for each isolate it could be concluded that both species exhibit a heterothallic mating system. They developed a PCR diagnostic to quickly identify the idiomorphs, and using this diagnostic test they investigated the distribution of mating-types for the U.S. populations of *A. flavus* and *A. parasiticus*. Global recombination rates appear to correlate with the distribution of mating-type idiomorphs within certain field populations – an equal distribution of *Mat1-1* and *Mat1-2* will yield higher incidences of recombination as well as yield greater diversity of aflatoxigenicity among individuals (Moore et al., 2009). Recombination generates more individual offspring with genomes that differ from both parents, meaning that sex in fungi may increase the number of vegetative compatibility groups (VCGs; Olarte et al., 2011; Moore et al., 2013a). When fungi like *A. flavus* out-cross, they overcome barriers such as vegetative incompatibility (likely to prevent cell death) and allow the exchange of novel genetic material between two fertile strains (Pál et al., 2007; Horn et al., 2009a). Supportive evidence for this circumvention of vegetative incompatibility exists through mating studies in *A. flavus* and *A. parasiticus* whereby the only successful pairings involved strains from different VCGs and opposite mating types (Horn et al., 2009a,b).

Investigations into the sexuality of three AF+ species (*A. parasiticus*, *A. flavus*, and *A. nomius*) eventually led to the discovery and characterization of their teleomorph states using the taxonomic nomenclature of genus *Petromyces* (Horn et al., 2009a,b, 2011). The teleomorph discoveries for the AF+ Aspergilli occurred in the laboratory, and there are some who might argue that experimental crosses in a controlled environment are not representative of natural field conditions. However, experimental field studies by Horn et al. (2013) demonstrated *A. flavus* sexuality in the field. Ears of corn were inoculated with compatible parental strains and later found to contain sclerotia. The sclerotia harvested from the ears of corn were not cleistothecium-bearing stromata; however, sclerotia collected from the corn and further incubated in and on non-sterile soil eventually contained cleistothecia. Reportedly, when these sclerotia fall onto the soil out-crossing is stimulated (Horn et al., 2013). Another teleomorph discovery is the evidence of hybridization reported between *A. flavus* and *A. parasiticus* (Worthington et al., 2011). This may mean that the biological species recognition concept, which defines a species as reproductively isolated (Taylor et al., 2006; Samson and Varga, 2009), cannot be applied to these aflatoxigenic Aspergilli.

*A. flavus*, *A. nomius,* and *A. parasiticus* exhibit self-incompatible (heterothallic) mating systems (Horn et al., 2009a,b, 2011). Of particular interest for *A. nomius* is that both mating-type idiomorphs may exist within a single strain, whereby certain isolates PCR-amplified as both *Mat1-1* and *Mat1-2*, and either idiomorph may be functional when out-crossing (Horn et al., 2011). A possible explanation for the presence of both mating-type idiomorphs may relate to the fact that *Aspergillus* species possess multi-nucleic conidiospores and hyphal cells, and are potentially heterokaryotic (Olarte et al., 2011; Runa et al., 2011). Olarte et al. (2011) examined progeny from out-crossing *A. flavus* strains that have either a full, partial, or absent AF gene cluster. In one generation of experimental crosses, most of the offspring exhibited recombinant genomes, having specific locus similarity to one or the other parent, while others were genetically distinct from both parents. They reported absence of cyclopiazonic acid (CPA) and AF loci in array Comparative Genomic Hybridization (aCGH) analysis for progeny, despite both parents having full and present CPA and AF clusters within their genomes, and yet the loci could be amplified using PCR. They posit that cryptic alleles influence genome variability, since some offspring were observed to amplify portions of the CPA and AF clusters when both parents exhibited the partial/absent AF cluster configuration (Olarte et al., 2011). Olarte’s research suggests that copy numbers of examined loci may influence the genomic condition observed, whereby low copy, ancestral alleles are often masked, but still present among heterokaryotic nuclei (Olarte et al., 2011).

## POTENTIAL IMPACTS OF SEXUAL RECOMBINATION ON *A. FLAVUS* BIOCONTROL

The effectiveness of pre-harvest biocontrol strategies using AF- *A. flavus *strains is based on their aggressiveness as competitors coupled with their inability to recombine with native AF+ strains, thereby preventing the re-acquisition of aflatoxigenicity (Ehrlich and Cotty, 2004; Abbas et al., 2011). Indeed, the biocontrol strains may be incapable of reinstated AF production, but not all of the offspring that result from their out-crossing will inherit the AF- phenotype. Through annual inundation of fields with biocontrol strains, the potential, over many generations, to increase the load of “super-competitors” with AF+ properties increases (Moore et al., 2013b). Given the possibility of obtaining multiple VCGs in a single generation, sexually promiscuous offspring could recombine with each other and further increase the population of AF+ individuals, thereby rendering the current biocontrol methodology ineffective in the future.

According to Olarte et al. (2011), the incidence of AF+ offspring observed was higher for crosses involving AF36 (58%) than for the Afla-Guard strain^®^ (36%). For offspring with an AF36 parent, replacement with a functional *pksA* gene can promote AF synthesis, but with the component strain in Afla-Guard^®^ (NRRL 21882) lacking the entire AF gene cluster, a simple replacement is less likely. Since the re-acquisition of AF cluster genes in offspring is less likely, use of a biocontrol strain lacking cluster genes such as NRRL 21882 would be preferable to one with an intact AF gene cluster. There is never complete inheritance of the AF- phenotype in the offspring of a biocontrol parent, unless both parents exhibit partial- or absent-cluster genotypes (Olarte et al., 2011). In field populations where AF+ strains are present, sex may yield toxigenic progeny. At this time, no studies have been reported that test colonization aggressiveness for progeny resulting from AF+ and biocontrol strain pairings, though there is evidence that AF levels are not significantly higher in the offspring from these out-cross events (Olarte et al., 2011). However, Moore et al. (2013a) reported that higher incidences of toxin diversity exist in actively recombining populations of *A. flavus* and *A. parasiticus*. For example, balancing selection in *A. flavus* seeks to maintain the AF- phenotype, but active recombination will alter the overall AF load of the population by reducing the numbers of AF- individuals (Moore et al., 2009, 2013a).

With regard to hybridization between *A. flavus* and *A. parasiticus*, perhaps the impact from their recombination may be important to agriculture beyond their mycotoxigenic potential. Firstly, *A. parasiticus* is predominately a soil inhabitant (Angle et al., 1982), whereas *A. flavus* is more ubiquitous (Zuluaga-Montero et al., 2010). Hybridization may allow *A. parasiticus* to alter or increase its niche through genetic modification. Hybrid offspring could exhibit far more diversity than recombinant offspring within each species (Olarte et al., 2011; Worthington et al., 2011). Perhaps, the most recently described AF+ “species” such as *A. parvisclerotigenus* and *A. minisclerotigenes* are actually hybrids, between *A. flavus* and B+G AF producers such as *A. parasiticus* or *A. nomius,* which have persisted due to selective advantages resulting from their hybridization events. Current molecular techniques will allow us to refute or support this hypothesis.

In the future, when seeking to use AF- *A. flavus* as pre-harvest biocontrol, there should be more diligence in researching the field ecology where biocontrol strain dispersal is intended for use or is currently in use. Specifically, the population density of native, potentially fertile AF+ species should be ascertained first by thorough field sampling; and additionally, the mating-type distribution of these native field strains should be determined since this will influence the stability of biocontrol (Moore et al., 2013a). If the field is predominately skewed to one mating type or the other, then using a biocontrol strain of the majority mating type could further restrict recombinant opportunities and slow the progression of both strain and toxin diversity (Moore et al., 2013a). In addition, any AF- strains sampled in a field should undergo extensive phenotypic and genomic investigations for consideration as biocontrol in the field where sampled (Moore et al., 2013a). Merely exhibiting the AF- phenotype is no longer a sufficient phenotypic criterion to warrant consideration of a strain as a candidate agent for biocontrol. Given its genotypic condition, the opportunity to generate AF+ offspring with a biocontrol strain such as NRRL 21882 is less likely than that with a strain such as AF36 (Olarte et al., 2011). Not only does NRRL 21882 exhibit the AF- phenotype because of it lacks AF cluster genes, but it also is incapable of producing CPA, another mycotoxin that is considered by some to have contributed to the severity of the Turkey X outbreak in the 1960s (Richard, 2008), while the AF36 biocontrol strain is AF- but produces CPA (Abbas et al., 2011), and CPA production has been observed in all offspring resulting from out-crossing with the AF36 parent (Olarte et al., 2011).

## FUNGAL SEX AND RECOMBINATION IN A CHANGING GLOBAL CLIMATE

There is increasing evidence that climate change is causing more unpredictability in global weather patterns. High heat and drought conditions stress plants and facilitate infection by aflatoxigenic species such as *A. flavus* (Scheidegger and Payne, 2003). Agricultural areas experiencing drought often suffer outbreaks of AF contamination. Moreover, diminished water availability limits the ability to irrigate and thereby mitigate the effects of drought (Kebede et al., 2012). At this time, incidences of AF outbreaks are most severe in tropical and sub-tropical areas (between latitudes 40°N and 40°S) around the world (Williams et al., 2004), and even temperate regions such as the United States Midwest are subject to occurrences of AF contamination. However, if current scientific reports are accurate, the average global surface temperature has been increasing by 0.15 °F each year since 1901 (United States Environmental Protection Agency [U.S.E.P.A], 2013). If temperatures continue to increase then the ideal climate for outbreaks of AF contamination will encompass more of our “temperate” agricultural regions and also become more frequent in occurrence. Therefore, it is imperative that as the research establishments continue to seek ways to control AF that researchers be aware of the potential impacts of climate change on the pathogenicity of AF+ fungi and the basic biology of these fungi. Sexual recombination often results from environmental stressors these fungi must overcome in order to adapt and survive. There is an extensive history of recombination in *A. flavus* (Moore et al., 2009). If global climate events assert constant negative pressures on AF+ Aspergilli, then this may accelerate the frequency of recombination in natural populations and lead to unfavorable outcomes for crop protection.

## CONCLUSION

The ultimate goal for using AF- *A. flavus* as biocontrol agents is long-term crop protection. Although biocontrol strains are reported to persist for years in inoculated fields (Cotty, 2013), current strategies require annual re-application of biocontrol strains. If the signature of the biocontrol strain is lost then perhaps recombination is to blame. Potentially, even a low rate of recombination between native AF+ fungi and introduced AF- fungi is significant when one considers future food safety. Generations from now, the aflatoxin problem may become more intractable because of the short-term method currently being used to prevent pre-harvest contamination. These fungi are – and have long been – sexually active. Their ability to evolve new phenotypes and genotypes via sexual recombination is a fact that cannot be ignored.

Sometimes, pathologists refer to plant: pathogen interactions as an evolutionary arms race (Anderson et al., 2010). Perhaps the same could be said for the use of non-aflatoxigenic strains for aflatoxin control. Hopefully, with continued research and understanding, we can maintain a consistent level of control without future risk of exacerbating the aflatoxin problem.

## Conflict of Interest Statement

The associate editor Perng-Kuang Chang declares that despite being in the same research area at the United States Department of Agriculture as the author GeromyMoore (Food and Feed Safety Research at Southern Regional Research Center), the review process was handled objectively and no conflict of interest exists.
